# Comprehensive Hand Evaluation Form: Feasibility of Merging the Most Common Hand-Specific Patient-Reported Outcome Measures

**DOI:** 10.3390/medicina62050929

**Published:** 2026-05-10

**Authors:** Matthias Holzbauer, Stefan Mathias Froschauer, Bernhard Oellinger, Paul Michael Schwarz, Sandra Feldler, Julian Alexander Mihalic, Tobias Gotterbarm

**Affiliations:** 1 Department of Orthopaedics and Traumatology, Kepler University Hospital GmbH, Krankenhausstrasse 9, 4020 Linz, Austria; paul.schwarz@kepleruniklinikum.at (P.M.S.); julian.mihalic@kepleruniklinikum.at (J.A.M.); tobias.gotterbarm@kepleruniklinikum.at (T.G.); 2 Medical Faculty, Johannes Kepler University Linz, Altenbergerstr. 69, 4040 Linz, Austria; stefan.froschauer@maz.at (S.M.F.); sandra.feldler@kepleruniklinikum.at (S.F.); 3 Department of Plastic, Aesthetic and Reconstructive Surgery, Kepler University Hospital, 4020 Linz, Austria

**Keywords:** DASH, equivalence testing, MHQ, PRWHE, outcome, validation

## Abstract

*Background and Objectives*: Patient-reported outcome measures (PROMs) are essential for evaluating outcomes in hand surgery, but the broad range of available instruments complicates selection and increases patient burden due to overlapping content. The Disabilities of the Arm, Shoulder and Hand (DASH), Michigan Hand Outcomes Questionnaire (MHQ), and Patient-Rated Wrist/Hand Evaluation (PRWHE) are the most frequently used PROMs. This study aimed to develop a merged instrument, the Comprehensive Hand Evaluation Form (CHEF), and to test whether CHEF-derived scores are equivalent to the original questionnaires. A secondary objective was to assess equivalence between pain ratings using an 11-item numeric rating scale (NRS) and a visual analogue scale (VAS). *Materials and Methods*: In this prospective study, adults with chronic atraumatic hand or wrist conditions completed the CHEF in the outpatient clinic and, three days later, the original DASH-G, MHQ-G, and PRWHE-G by mail. Equivalence was evaluated using two one-sided tests with margins set at half the minimal clinically important difference. Completion times were recorded. *Results*: Of the 100 patients, 57 could be included in the final analysis. Equivalence between CHEF-derived and original scores was demonstrated for PRWHE-G (mean difference −2.0; 90% CI −4.4 to 0.4 within ±7). Equivalence was not demonstrated for DASH-G (mean difference −3.5; 90% CI −5.7 to −1.3; margin ±5) or MHQ-G (mean difference 13.3; 90% CI 10.6 to 15.9; margin ±4.5). Thus, equivalence was achieved for one of three instruments. CHEF completion time was significantly shorter than the combined original questionnaires (median 10 vs. 15 min; *p* < 0.0001). For pain assessment, equivalence between the numeric rating scale and the visual analogue scale was observed at rest but not during activity. *Conclusions*: CHEF reduced completion time but achieved score equivalence only for PRWHE-G. These findings suggest that integrated PROM approaches may reduce burden, but do not consistently preserve equivalence across instruments.

## 1. Introduction

Patient-reported outcome measures (PROMs) are widely used to evaluate treatment outcomes from the patient’s perspective and to compare therapeutic interventions and surgical procedures. In 2021, Woythal et al. identified 73 PROMs used to assess hand conditions [1]. These instruments can be classified into generic questionnaires, such as the Short Form 36 [2] or European Quality of Life 5 Dimensions [3], and disease-specific questionnaires, such as the Boston carpal tunnel Questionnaire [4], the Australian Canadian Osteoarthritis Hand Index [5], and the Unité Rhumatologique des Affections de la Main for Dupuytren’s disease [6]. Additional categories include symptom-specific questionnaires, such as the Cold Intolerance Symptom Severity [7], and region-specific instruments. Region-specific instruments represent the most commonly used measures in hand surgery, with the Disabilities of the Arm, Shoulder, and Hand (DASH) questionnaire being the most frequently used instrument [8,9]. This widespread use is reflected by the fact that the DASH has been translated and validated in a large number of languages and is currently available in at least 41 countries across Europe [10]. While the DASH, which was later adapted into a shorter version, the QuickDASH [11], assesses function across the entire upper extremity, more regionally specific questionnaires are also available. In descending order of citation frequency [8,9], the DASH is followed by the Patient-Rated Wrist Evaluation (PRWE) [12], the Michigan Hand Outcomes Questionnaire (MHQ) [8], which is also available in a shorter version, the BriefMHQ [13].

The DASH comprises 30 items rated on a 5-point Likert scale assessing physical function (21 items), symptoms (6 items), and social role (3 items), yielding a total score from 0 (no impairment) to 100 (maximum impairment) [14]. The MHQ consists of six subscales—overall hand function, activities of daily living, work performance, pain, aesthetics, and patient satisfaction—rated on 5-point Likert scales and normalized to scores ranging from 0 (worst) to 100 (best) for each subscale and the total score [15]. The PRWE includes a pain subscale (5 items) and a function subscale (10 items) rated on an 11-point Likert scale, resulting in a total score from 0 (no pain or difficulty) to 100 (maximum pain or difficulty) [12]. In 2004, the PRWE was expanded to include hand injuries, maintaining the same format and resulting in the Patient-Rated Wrist/Hand Evaluation (PRWHE) [16]. All three questionnaires have been translated and validated in multiple languages and are available in German as DASH-G, MHQ-G, and PRWE-G [17,18,19].

Patient-centred assessment of treatment effects is increasingly important in hand surgery to provide evidence of therapeutic benefit [20]. However, there is no consensus on which PROM should be used for specific conditions [21,22,23]. Given this broad range of available tools, healthcare professionals and researchers often face difficulties in selecting the most appropriate PROM for hand and wrist conditions. Consequently, variability in PROM selection hampers comparison across studies and limits the ability to pool data for systematic reviews and meta-analyses [24]. A recent international consensus initiative by the International Consortium for Health Outcomes Measurement identified numerous outcome instruments for assessing domains such as pain, hand function, and quality of life in patients with hand and wrist conditions [25]. Within this initiative, five measurement tracks (the thumb track, the finger track, the wrist track, the nerve track, and the severe trauma track) for broadly defined condition groups were established, accompanied by recommendations regarding outcome instruments and time points for measurement [25]. The large variety of instruments illustrates the heterogeneity of outcome assessment in hand surgery and highlights the need for approaches that reduce redundancy while preserving comparability across studies.

In their scoping review, Woythal et al. identified 179 nonredundant items across 73 hand-specific PROMs after removing linguistic and grammatical overlap, and categorized them into impairment (symptoms), activity (activities of daily living and sports/recreational activities), and participation (psychosocial domains) [1]. Building on this concept, the present study aimed to merge the most commonly used hand-specific PROMs—DASH-G, MHQ-G, and PRWHE-G—into a single instrument, the Comprehensive Hand Evaluation Form (CHEF). This pilot study tests the hypothesis that scores derived from the CHEF are equivalent to those obtained from the original questionnaires. The rationale is to reduce patient burden by avoiding redundant questions and to shorten completion time, while enabling simultaneous calculation of three PROM scores from one questionnaire. From a clinical and research perspective, this approach could facilitate broader outcome assessment and improve comparability across studies. As pain is a key symptom in hand surgery, a secondary objective was to investigate the equivalence of pain assessment using a numeric rating scale and a visual analogue scale.

Therefore, the aim of this study was to evaluate the CHEF questionnaire in patients with chronic hand and wrist conditions and to test the hypothesis that CHEF-derived outcome scores are equivalent to those obtained from established patient-reported outcome measures for hand function.

## 2. Materials and Methods

The regional ethics committee approved this prospective trial prior to study initiation (No.: EK-1320/2020). Written informed consent was obtained from all participants.

### 2.1. Development of the Comprehensive Hand Evaluation Form (CHEF)

The Comprehensive Hand Evaluation Form (CHEF) was developed through a structured content-integration process based on the German versions of the three most frequently used region-specific hand PROMs: DASH-G, MHQ-G, and PRWHE-G. These instruments have undergone formal cross-cultural adaptation and validation for German-speaking populations [17,18,19]. The objective was to construct a single composite instrument that would preserve the conceptual domains and scoring logic of the original questionnaires while minimizing redundant content. Geographical and cultural adaptation had already been addressed through the validated German translations of the original questionnaires [17,18,19]. Since the CHEF adopted these items without modification, no additional linguistic or cultural adaptation was performed during their integration into the CHEF.

Initially, all items from the DASH-G (30 items), MHQ-G (including six subscales), and PRWHE-G (15 items) were systematically reviewed by the lead author (MH). Each item was categorized according to its conceptual domain. Overlapping or semantically equivalent items across instruments were identified through direct comparison of item wording, response format, and underlying construct.

Items assessing comparable constructs (e.g., opening a jar, carrying objects, pain during activity) were consolidated into single representative CHEF items. Where instruments differed in response scaling (e.g., 5-point Likert scale vs. 11-point numeric rating scale), the original response format was retained when necessary to allow valid back-calculation of the respective instrument scores. In cases where direct item equivalence was not possible, transformation algorithms were predefined to ensure score compatibility.

The resulting CHEF instrument was organized into six domains reflecting the combined content structure of the original questionnaires:

1. Activities of daily living.
2. Pain and physical symptoms.
3. Frequency of work-related limitations.
4. Functional performance of the affected hand.
5. Satisfaction with hand function.
6. Social and aesthetic impact.

Each CHEF item was explicitly annotated to indicate its contribution to one or more of the embedded questionnaires (DASH-G, MHQ-G, PRWHE-G). Where necessary, arithmetic means of grouped CHEF items were calculated to represent composite items in the original instruments (e.g., PRWHE personal care domain, MHQ pain item). Detailed scoring formulas for reconstructing each original questionnaire score from CHEF responses were predefined and are provided in the Supplementary Materials, which also include the full CHEF questionnaire.

Because the original PROMs differ in scaling direction, number of response options, and score normalization procedures, dedicated conversion formulas were developed prior to study initiation. These algorithms accounted for:

- Directional inversion of scales where required (e.g., function scored as impairment vs. capability).
- Rescaling between 5-point Likert and 11-point numeric formats.
- Normalization procedures specific to each instrument.
- Handling of missing items in accordance with the respective validation studies.

The predefined formulas enabled computation of CHEF[DASH-G], CHEF[MHQ-G], and CHEF[PRWHE-G] scores that were methodologically aligned with the scoring manuals of the original instruments.

The preliminary CHEF version underwent internal review by two additional study investigators with clinical expertise in hand surgery. The review focused on linguistic clarity, structural coherence, and preservation of the original constructs. Minor modifications were implemented to improve comprehensibility and logical sequencing of items without altering conceptual equivalence.

No patient pre-testing or cognitive debriefing was conducted prior to the pilot study, as the primary objective was equivalence testing rather than independent validation of a novel construct.

Prior to reproduction and modification of the instruments, written permission was obtained from the respective copyright holders of DASH-G, MHQ-G, and PRWHE-G. Applicable licensing fees were paid via the publisher’s RightsLink copyright clearance system.

### 2.2. Study Population

Consecutive adult patients aged ≥18 years presenting with atraumatic hand conditions were enrolled.

The following inclusion criteria were defined:

- Isolated, chronic hand or wrist condition (e.g., tendinitis, arthritis, or nerve compression syndromes).
- No therapeutic interventions at the index visit (e.g., prescription of nonsteroidal anti-inflammatory drugs, oral or injected steroids, immobilization, or referral to hand therapy), ensuring that the condition was expected to remain stable during the subsequent two-week period.
- Native-level proficiency in German to ensure linguistic and cultural consistency in questionnaire interpretation.

The exclusion criteria were as follows:

- Acute traumatic conditions.
- History of hand surgery to either hand within recent years.
- Insufficient German proficiency.
- Cognitive impairment precluding informed consent.

Patients whose questionnaires did not meet the predefined minimum completeness criteria were excluded from the per-protocol analysis.

Eligibility was assessed after completion of the clinical consultation to avoid influencing medical decision-making. After consent, demographic and clinical data were recorded. Patients then completed the CHEF in the outpatient waiting area at their own pace, without study personnel present. Completion time was documented, and the questionnaire was sealed in an envelope until analysis.

Three working days later, patients received by mail the original DASH-G, MHQ-G, and PRWHE-G questionnaires, along with a cover letter and return envelope. Both the CHEF and postal questionnaires included visual analogue scales (VAS) for pain at rest and during activity, consisting of a 10 cm line anchored by “no pain” and “worst pain imaginable.”

Patients were instructed to complete the questionnaires independently, record the time required, and rank the four questionnaires from 1 (best) to 4 (worst) regarding clarity and comprehensibility. They were also asked whether they noticed similar or identical questions across questionnaires, whether this redundancy caused discomfort, and whether completing questionnaires in the outpatient clinic was uncomfortable (yes/no). Completed forms were returned by mail for analysis.

### 2.3. Statistical Methods

All data from the paper-based questionnaires were digitized, and a spreadsheet was used to calculate the CHEF[DASH-G], CHEF[MHQ-G], and CHEF[PRWHE-G] scores according to the predefined formulas derived from the individual CHEF items.

Subsequently, the minimum requirements for the analysis of each questionnaire were checked. For the DASH-G, no more than three missing items were allowed [17]. For each MHQ-G subscale, fewer than 50% of items were permitted to be missing [15]. For the PRWHE-G, John et al. recommended that no more than one third of the items per subscale be missing, corresponding to a minimum of three responses for the pain subscale and seven for the function subscale [18]. For the MHQ-G and PRWHE-G, missing values within a subscale were imputed using the mean of the remaining items of that subscale [15,18]. The same rules were applied to the corresponding CHEF-derived scores (CHEF[DASH-G], CHEF[MHQ-G], and CHEF[PRWHE-G]).

Standard descriptive statistics were used. For continuous variables, normality was assessed using the Kolmogorov–Smirnov test with Lilliefors correction. Normally distributed data are presented as mean (standard deviation, SD), and non-normally distributed data as median (interquartile range, IQR). Ordinal variables are presented as median (IQR), and nominal variables as absolute frequencies.

The primary outcome measures were the CHEF-derived subscores (CHEF[DASH-G], CHEF[MHQ-G], CHEF[PRWHE-G]) and the corresponding original questionnaire scores (DASH-G, MHQ-G, PRWHE-G).

Equivalence testing was performed using the two one-sided tests (TOST) procedure. The upper and lower equivalence margins were defined as half of the minimal clinically important difference (MCID) reported in the literature. Accordingly, an MCID of 10 was used for the DASH score [26,27] and 14 for the PRWHE [26]. Although MCIDs have been reported for individual MHQ subscales [28,29], an MCID of 9 for the MHQ total score was applied in this study [28].

Thus, the exploratory hypothesis was that equivalence between CHEF-derived scores and the original questionnaires could be assumed if the differences between CHEF[DASH-G], CHEF[MHQ-G], and CHEF[PRWHE-G] and their corresponding original scores lay within the predefined equivalence margins.

Randall et al. reported an MCID of 1.6 for VAS pain scores in hand conditions [30]. The first two items of the PRWHE-G represent an 11-point numeric rating scale (NRS) for pain at rest and during activity. These NRS scores (referred to as CHEF[NRS rest/activity] and PRWHE[NRS rest/activity]) were tested for equivalence with the corresponding VAS scores, both for values obtained from the CHEF and from the postal questionnaires. In addition, agreement over time between NRS and VAS measurements was assessed.

The significance level was set at an α of 0.05. Equivalence was concluded if the two-sided 90% confidence interval of the mean difference lay entirely within the predefined equivalence margins, corresponding to the TOST procedure with an overall α of 0.05 [31]. Graphical illustration of agreement and equivalence between the CHEF-derived and original questionnaire scores, as well as between VAS and NRS measurements, was performed using a Bland–Altman plot.

Depending on data distribution, completion times were compared using paired t-tests or Wilcoxon signed-rank tests. A Friedman test was used to compare clarity and comprehensibility among the four questionnaires, with post hoc pairwise Wilcoxon signed-rank tests performed if significant.

Because CHEF is a newly developed instrument and no prior data were available to estimate the expected standard deviation required for an a priori power calculation for equivalence testing, the study was designed as a pragmatic pilot study. A target sample size of 100 patients was chosen based on statistical consultation, allowing for an anticipated dropout rate of at least 20%.

## 3. Results

Between January and June 2021, 100 patients were enrolled in this prospective study. Of these, 78 returned the return envelope containing the original questionnaires. The minimum requirements for analysis were not met by 21 patients (27%), due to insufficient data for one or more questionnaires: CHEF[DASH-G] (*n* = 13), CHEF[MHQ-G] (*n* = 8), CHEF[PRWHE-G] (*n* = 6), MHQ-G (*n* = 6), DASH-G (*n* = 5), and PRWHE-G (*n* = 4). Consequently, 57 patients were included in the final analysis (24 women and 33 men; median age 53 years, IQR 18). The diagnoses of the included conditions are listed in Table 1. 33 and 22 conditions affected the left and right hands, respectively. In addition, one patient with bilateral trapeziometacarpal osteoarthritis and one patient with bilateral carpal tunnel syndrome were included. 51 patients were right-hand dominant, and six patients were left-hand dominant. The reported comorbidities included arterial hypertension (*n* = 10), diabetes mellitus (*n* = 4), rheumatic disease (*n* = 4), osteoporosis (*n* = 4), depression (*n* = 3), and neurological disorders (*n* = 2). No patients reported gout. Twelve patients were current smokers, and five reported regular alcohol consumption. The highest educational attainment reported was an apprenticeship (*n* = 41), an upper secondary school diploma (*n* = 10), and a university degree (*n* = 5). 33 patients reported their occupation: 23 patients worked in non-manual occupations (employees, civil servants, psychologists), nine in manual occupations (manual workers, farmers, professional drivers, police officers), and one patient was self-employed.

Equivalence testing using the TOST was concluded when the two-sided 90% confidence interval (CI) of the mean difference lay entirely within the predefined margins (±5 for DASH-G, ±7 for PRWHE-G, and ±4.5 for the MHQ total score).

**Figure 1:**
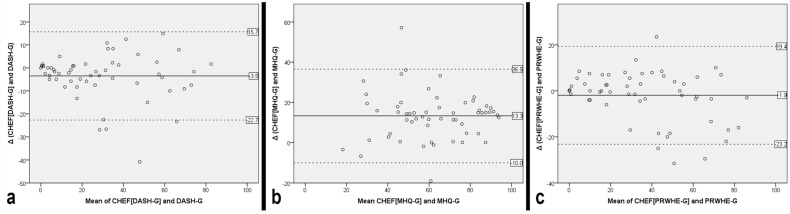
Bland–Altman plots illustrating agreement between CHEF-derived and original questionnaire scores for DASH-G (**a**), MHQ-G total score (**b**), and PRWHE-G (**c**).

For DASH-G, the mean difference between CHEF[DASH-G] and DASH-G was −3.5 (SD 9.8), with a 90% CI of −5.7 to −1.3. Because this interval extended beyond the lower equivalence margin of −5, equivalence could not be established. For the MHQ total score, the mean difference between CHEF[MHQ-G] and MHQ-G was 13.3 (SD 11.9), with a 90% CI of 10.6 to 15.9. As the entire interval lay outside the equivalence margins of ±4.5, equivalence was clearly not demonstrated. For PRWHE-G, the mean difference between CHEF[PRWHE-G] and PRWHE-G was −2.0 (SD 10.8), with a 90% CI of −4.4 to 0.4. As this interval lay entirely within the equivalence margins of ±7, equivalence was supported. Overall, equivalence was demonstrated for PRWHE-G, but not for DASH-G or the MHQ-G total score. Bland–Altman plots for all three questionnaires are shown in Figure 1.

**Figure 2:**
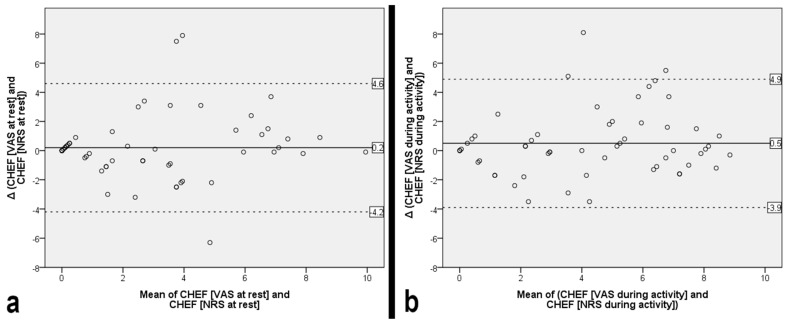
Bland–Altman plot illustrating agreement between visual analogue scale (VAS) and numeric rating scale (NRS) pain scores at rest (**a**) and during activity (**b**).

Pain assessment results are presented in Table 2. The agreement between pain ratings obtained using the VAS and NRS is illustrated by a Bland–Altman plot in Figure 2, demonstrating equivalence for pain evaluation at rest, whereas no equivalence was observed during activity.

The median completion time for the CHEF was significantly shorter than for the three original questionnaires combined (10 [IQR 7] vs. 15 [IQR 11] minutes; Wilcoxon signed-rank tests, *p* < 0.0001). However, only 37 of 51 patients (73%) subjectively perceived the CHEF as quicker to complete. All respondents (*n* = 53) reported that the original questionnaires contained similar or identical questions, and 17 of 54 patients (30%) indicated discomfort with this redundancy. Five of 54 patients (9%) reported discomfort completing questionnaires in the outpatient clinic in general.

A Friedman test was performed to compare perceived clarity and comprehensibility of the four questionnaires (CHEF, DASH-G, PRWHE-G, and MHQ-G) in 41 and 43 patients, respectively. Significant overall differences were observed for both clarity (*p* = 0.0019) and comprehensibility (*p* = 0.0008). Regarding clarity, PRWHE-G was rated significantly worse than CHEF (*p* = 0.024), DASH-G (*p* = 0.012), and MHQ-G (*p* = 0.004). Regarding comprehensibility, DASH-G was rated significantly better than CHEF (*p* = 0.023) and PRWHE-G (*p* = 0.0039), and PRWHE-G was rated significantly worse than MHQ-G (*p* = 0.018).

## 4. Discussion

The main finding of this study is that the newly developed CHEF questionnaire significantly reduced completion time compared with the three original PROMs. However, score equivalence was demonstrated only for PRWHE-G and not for DASH-G or the MHQ-G total score when applying predefined equivalence margins based on half of the respective MCIDs. Consequently, the hypothesis that CHEF-derived scores for DASH-G, MHQ-G, and PRWHE-G would be equivalent to those obtained from the original questionnaires could not be confirmed.

Completion of the CHEF required significantly less time than completion of the three original questionnaires combined (median 10 vs. 15 min), although only 73% of patients subjectively perceived the CHEF as faster. Despite voluntary participation and explicit instructions to complete all items, the return rate was 78%, and 27% of returned questionnaires were excluded because the minimum completeness criteria were not met. Dropout occurred least frequently for CHEF[PRWHE-G] and PRWHE-G, which may reflect the smaller number of items in this instrument. A Cochrane review reported higher response rates with shorter questionnaires [32], supporting the notion that response burden influences completion. Response fatigue is known to contribute to missing data, bias, and reduced engagement [33].

The order of questionnaire administration may also have influenced response fatigue. The MHQ-G, which appeared last within the CHEF and was also completed last in the postal set, showed the largest mean difference between CHEF-derived and original scores (13.3 ± 11.9), compared with −3.5 ± 9.8 for DASH-G and −2.0 ± 10.8 for PRWHE-G. Overall, the second postal assessment resulted in worse scores in all original questionnaires compared to the CHEF subscores. Notably, the deterioration in MHQ-G exceeded its MCID of nine points, suggesting a clinically relevant effect that may reflect increased fatigue during the longer postal assessment, which required approximately 1.5 times more completion time than the CHEF. Consistent with this interpretation, many MHQ-G subscores were answered with identical response options toward the end of both the CHEF and postal questionnaires. Furthermore, at least 30% of patients reported discomfort with answering similar or identical questions, whereas only 9% felt uncomfortable completing questionnaires in the outpatient clinic in general.

One strategy to address response burden is computerized adaptive testing (CAT), which dynamically selects items based on prior responses to optimize measurement precision while minimizing the number of questions [33,34]. CAT has been shown to generate more precise DASH scores than the QuickDASH using fewer items (mean 8.5) [35]. Similarly, a CAT algorithm for the PRWHE has reduced the median number of required items to three [24].

With respect to pain assessment, the VAS and the 11-point NRS are the most commonly recommended formats [36]. Our findings support previous evidence that these scales are not mathematically equivalent or interchangeable, with VAS values tending to be higher [36,37]. In this study, differences between VAS and NRS scores for pain during activity exceeded the predefined equivalence margin, precluding equivalence. In contrast, VAS and NRS values obtained from CHEF and postal questionnaires were equivalent, supporting the internal consistency of measurements across settings. These results underscore the importance of precise methodological reporting of pain assessment methods in hand surgery research [36].

Another important aspect when interpreting PROMs is the influence of geographical and cultural factors. Differences in language, healthcare systems, and daily functional demands may affect how patients perceive and report hand function. A recent analysis of PROM availability across Europe identified 47 countries with 41 official languages and demonstrated considerable variability in the availability of commonly used hand outcome measures. The DASH questionnaire was available in 41 countries (87%), whereas translations of the PRWE, BCTQ, and MHQ were available in 66%, 64%, and 43% of countries, respectively [10]. In the studies evaluated in the review by Iskander et al., PROMs were most frequently reported by authors from the United States (*n* = 58), the Netherlands (*n* = 37), the United Kingdom (*n* = 31), Sweden (*n* = 21), and Germany (*n* = 20) [8]. These findings highlight the importance of cross-cultural adaptation and validation when applying PROMs across different linguistic and cultural settings.

Certain limitations should be acknowledged when interpreting this study. The study population was diagnostically heterogeneous and comorbidities were recorded only descriptively; therefore, instrument performance may differ across specific conditions. The fixed order of administration, with CHEF always completed before the original questionnaires, and the different modes of completion (in-clinic vs. postal) may have introduced order and context effects that cannot be disentangled from true instrument differences. Furthermore, only cross-sectional agreement was assessed; reliability, responsiveness, and construct validity of the CHEF were not evaluated in this pilot study, as equivalence with the original questionnaires first needed to be established. Although the mathematical conversion of the CHEF[PRWHE-G] functional scale from a 5-point to an 11-point format was considered a potential source of bias, PRWHE-G was the only instrument for which equivalence was demonstrated, despite being rated least clear and comprehensible by patients. Furthermore, the CHEF was developed and evaluated in a German-speaking population. While the items of the CHEF were extracted from validated versions of the translated original questionnaires, the cultural and regional factors may influence how patients perceive and report hand function; therefore, the applicability of the instrument in other linguistic or cultural settings requires further evaluation.

## 5. Conclusions

The CHEF significantly reduced completion time compared with the three original PROMs but achieved score equivalence only for PRWHE-G. The lack of equivalence for DASH-G and MHQ-G may be attributable to response fatigue associated with longer questionnaires. Although CHEF did not achieve interchangeability with all underlying PROMs, this novel approach highlights the potential of integrating commonly used instruments into a single tool to reduce response burden. Future developments, particularly CAT-based approaches, may further enable tailored outcome assessment while minimizing patient fatigue and preserving measurement precision.

## Figures and Tables

**Table 1:** Diagnoses of the included patients.

Diagnosis	*n*
Carpal tunnel syndrome	15
Dupuytren’s disease	15
Trigger finger	11
Dorsal wrist ganglion	5
Trapeziometacarpal joint osteoarthritis	2
Radiocarpal joint osteoarthritis	2
De Quervain’s disease	1
Scaphoid nonunion advanced collapse (SNAC wrist)	1
Kienböck’s disease	1
Neuroma (proper digital nerve or ulnar nerve)	1
Chronic triangular fibrocartilage complex (TFCC) rupture	1
Heberden’s osteoarthritis	1

**Table 2:** Equivalence and Agreement Between Pain Scores Assessed by Visual Analogue Scale (VAS) and Numeric Rating Scale (NRS) Using CHEF and PRWHE * equivalence was supported because both intervals lay fully within the equivalence margins of ±0.8 (defined as half of the minimal clinically important difference reported by Randall et al. [30]).

Parameters for Pain	N	Absolute Outcomes	Difference	90% Confidence Interval
CHEF [VAS at rest] – CHEF [NRS at rest]	56 56	3.0 (SD 3.0) 2.8 (SD 2.8)	0.2 (SD 2.3)	[−0.3, 0.7] *
CHEF [VAS during activity] – CHEF [NRS during activity]	56 56	4.5 (SD 3.1) 4.0 (SD 2.8)	0.5 (SD 2.2)	[0.0, 1.0]
VAS at rest – PRWHE [NRS at rest]	56 56	2.7 (SD 2.6) 2.4 (SD 2.6)	0.3 (SD 1.8)	[−0.1, 0.7] *
VAS during activity – PRWHE [NRS during activity]	57 57	4.6 (SD 3.0) 4.0 (SD 3.0)	0.6 (SD 2.2)	[0.1, 1.1]
CHEF [VAS at rest] – VAS at rest	57 57	3.0 (SD 3.0) 2.7 (SD 2.6)	0.3 (SD 1.7)	[−0.4, 0.7] *
CHEF [VAS during activity] – VAS during activity	57 57	4.6 (SD 3.1) 4.6 (SD 3.0)	0 (SD 1.6)	[−0.4, 0.3] *
CHEF [NRS at rest] – PRWHE [NRS at rest]	55 55	2.8 (SD 2.8) 2.4 (SD 2.7)	0.4 (SD 1.5)	[0.2, 0.7] *
CHEF [NRS during activity] – PRWHE [NRS during activity]	56 56	4.0 (SD 2.8) 4.0 (SD 3.0)	0.0 (SD 2.0)	[−0.4, 0.5] *

## Data Availability

The datasets analyzed during the current study are available from the corresponding author upon reasonable request.

## Supplementary Materials

The following supporting information can be downloaded at https://www.mdpi.com/article/10.3390/medicina62050929/s1. Comprehensive Hand Evaluation Form.

## Abbreviations

The following abbreviations are used in this manuscript:

CHEF	Comprehensive Hand Evaluation Form
DASH	Disabilities of the Arm, Shoulder and Hand
IQR	Interquartile Range
MHQ	Michigan Hand Outcomes Questionnaire
NRS	Numeric rating scale
PROM	Patient-reported outcome measures
PRWHE	Patient-Rated Wrist/Hand Evaluation
SD	Standard Deviation
TOST	Two one-sided tests
VAS	Visual analogue scale
