# Supplementary material for: Comprehensive Hand Evaluation Form: Feasibility of Merging the Most Common Hand-Specific Patient-Reported Outcome Measures
Source: Medicina (Kaunas). 2026 May 10;62(5):929. doi: 10.3390/medicina62050929 (PMC13208409; doi:10.3390/medicina62050929)
Supplement: Supplementary file 1 [file medicina-62-00929-s001.zip › medicina-4185891-supplementary.pdf]

Comprehensive Hand Evaluation Form

This version of the Comprehensive Hand Evaluation Form (CHEF) includes a comment column indicating which CHEF item was used to calculate each of the three incorporated questionnaires: the Disabilities of the Arm, Shoulder, and Hand (D), the Michigan Hand Outcomes Questionnaire (M), and the Patient-Rated Wrist Evaluation (P). Moreover, the relevant formulas for transforming the individual items within each questionnaire have been added after each subsection.

1. *Alltagsfunktionen*

**Nr**		**Keine Schwierig-keiten**	**Geringe Schwierig-keiten**	**Mäßige Schwierig-keiten**	**Deutliche Schwierig-keiten**	**Nicht möglich**	Comment
1	Ein neues oder fest verschlossenes Konservenglas öffnen	1	2	3	4	5	D,M
2	Schreiben	1	2	3	4	5	D
3	Aufheben einer Münze	1	2	3	4	5	M
4	Einen Schlüssel im Schloss drehen	1	2	3	4	5	D,M
5	Mit der betroffenen Hand einen Türknauf drehen	1	2	3	4	5	P[1],M
6	Ein Glas Wasser halten	1	2	3	4	5	M
7	Eine Mahlzeit zubereiten	1	2	3	4	5	D
8	Eine Bratpfanne halten	1	2	3	4	5	M
9	Geschirr spülen	1	2	3	4	5	M
10	Eine schwere Tür aufstoßen	1	2	3	4	5	D
11	Einen Gegenstand in ein Regal über Kopfhöhe stellen	1	2	3	4	5	D
12	Schwere Hausarbeit durchführen (z.B. Gardinen mit der Hand waschen, Böden wischen, kleine Reparaturen)	1	2	3	4	5	D,P[1]
13	Gartenarbeit ausführen	1	2	3	4	5	D
14	Ein Bett machen	1	2	3	4	5	D
15	Eine Einkaufstüte oder eine Aktentasche tragen	1	2	3	4	5	D,M
16	Einen schweren Gegenstand (über 5 kg) tragen	1	2	3	4	5	D,P[1]
17	Eine Glühbirne in einer Deckenlampe tauschen	1	2	3	4	5	D
18	Ihre Haare waschen oder föhnen	1	2	3	4	5	D,P[2],M
19	Ihren Rücken waschen	1	2	3	4	5	D,P[2]
20	Mit der betroffenen Hand Toilettenpapier nutzen	1	2	3	4	5	P[1]
21	Einen Pullover anziehen	1	2	3	4	5	D,P[2]
22	Ein Hemd oder eine Bluse zuknöpfen	1	2	3	4	5	P[1],M
23	Schuhe zuknöpfen/Knoten Knüpfen	1	2	3	4	5	M
24	Die betroffene Hand nutzen, um von einem Stuhl aufzustehen	1	2	3	4	5	P[1]
25	Ein Messer zum Schneiden von Brot oder Fleisch verwenden	1	2	3	4	5	D,P[1],M
26	Arbeit (Beruf oder Alltagstätigkeiten)	1	2	3	4	5	P[1]

27	Freizeitbeschäftigung durchführen, die wenig Mühen erfordern (z.B. Kartenspielen, Stricken, etc.)	1	2	3	4	5	D,P[3]
28	Freizeitbeschäftigung durchführen, die etwas mehr Kraft oder Druck durch Ihre Hände erfordern (z.B. Kegeln, Tennis, einen Hammer benutzen)	1	2	3	4	5	D,P[3]
29	Freizeitbeschäftigung durchführen, bei denen Ihre Arme frei bewegt werden (z.B. Federball spielen)	1	2	3	4	5	D,P[3]
30	Beim Verwenden von Transportmitteln (von einem Ort zum anderen gelangen)	1	2	3	4	5	D
31	Sexuelle Aktivitäten	1	2	3	4	5	D

[1] To convert the CHEF score into the PRWHE, the following formula must be applied:

$$PRWHE_{score} = 2{,}5 * (CHEF_{score} - 1)$$

[2] For these three items, the arithmetic mean is calculated, and then the formula described in [1] is applied to represent the PRWHE item ‘Persönliche Körperpflege (Anziehen, Waschen)’

[3] For these three items, the arithmetic mean is calculated, and then the formula described in 1 is applied to represent the PRWHE item ‘Freizeitaktivitäten’.

2. *Schmerz und körperliche Beschwerden*

**Nr**		**Keine**		**Gering**		**Mäßig**		**Starke**		**Schlimmste**			Anm
1	Ruheschmerzen in den Händen	0	1	2	3	4	5	6	7	8	9	10	D[4],P,M[5]
2	Schmerzen bei der Ausübung bestimmter Tätigkeiten	0	1	2	3	4	5	6	7	8	9	10	D[4]
3	Schmerzen bei Tätigkeiten mit wiederholter Bewegung	0	1	2	3	4	5	6	7	8	9	10	P
4	Schmerzen beim Heben eines schweren Gegenstandes	0	1	2	3	4	5	6	7	8	9	10	P
5	Wenn die Schmerzen am stärksten sind	0	1	2	3	4	5	6	7	8	9	10	P,M[5]
6	Schwäche in den Händen	0	1	2	3	4	5	6	7	8	9	10	D[4]
7	Kribbeln in den Händen	0	1	2	3	4	5	6	7	8	9	10	D[4]
8	Steifigkeit der Hände	0	1	2	3	4	5	6	7	8	9	10	D[4]
9	Hatten Sie in den letzten Wochen Schlafstörungen wegen Schmerzen in Ihren Händen?	0	1	2	3	4	5	6	7	8	9	10	D[4]

[4] To convert the CHEF score into the DASH, the following formula must be applied:

$$DASH_{score} = 0{,}4 * CHEF_{score} + 1$$

[5] For these two items, the arithmetic mean is calculated, and then the following formula is applied to represent the MHQ item ‘Bitte beschreiben Sie den Schmerz in Ihren Händen‘.

$$MHOQ_{score} = 0{,}4 * CHEF_{score} + 1$$

3. *Häufigkeit der Einschränkung in den letzten 4 Wochen im Arbeitsalltag (Beruf, Hausarbeit, Schule etc.)*

Nr		Nie	Selten	Manchmal	Oft	Immer	Anm
1	Schmerzhäufigkeit	1	2	3	4	5	M,P[5]
2	Wie oft waren Sie aufgrund Ihrer Beschwerden unfähig, Ihre Arbeit zu verrichten?	1	2	3	4	5	M
3	Wie oft waren Sie aufgrund Ihrer Beschwerden gezwungen, Ihren Arbeitsalltag zu verkürzen?	1	2	3	4	5	M
4	Wie oft mussten Sie es bei der Arbeit aufgrund Ihrer Beschwerden ruhiger angehen lassen?	1	2	3	4	5	M
5	Wie oft konnten Sie aufgrund Ihrer Beschwerden bei der Arbeit weniger als geplant leisten?	1	2	3	4	5	M
6	Wie oft haben Sie aufgrund Ihrer Beschwerden länger bei der Arbeit gebraucht?	1	2	3	4	5	M
7	Wie oft stören Ihre Schmerzen in der Hand Ihren Schlaf?	1	2	3	4	5	M
8	Wie oft behindern Sie die Schmerzen in Ihren Händen bei Ihren täglichen Aktivitäten?	1	2	3	4	5	M
9	Wie oft machten Sie die Schmerzen in Ihren Händen unglücklich?	1	2	3	4	5	M

[5] To convert the CHEF score into the PRWHE, the following formula must be applied:

$$PRWHE_{score} = 2{,}5 * (CHEF_{score} - 1)$$

4. *Funktion der betroffenen Hand*

Nr		Sehr gut	Gut	Mittelmäßig	Schlecht	Sehr Schlecht	
1	allgemeine Funktion der Hand	1	2	3	4	5	M
2	Beweglichkeit der Finger	1	2	3	4	5	M
3	Beweglichkeit des Handgelenks	1	2	3	4	5	M
4	Kraft der Hand	1	2	3	4	5	M
5	Sensibilität (das Gefühl) der Hand	1	2	3	4	5	M

5. *Zufriedenheit mit der betroffenen Hand*

Nr		Sehr zufrieden	Zufrieden	Weder zufrieden noch unzufrieden	Etwas unzufrieden	Sehr unzufrieden	
1	allgemeine Funktion der Hand	1	2	3	4	5	M
2	Beweglichkeit der Finger	1	2	3	4	5	M
3	Beweglichkeit des Handgelenks	1	2	3	4	5	M
4	Kraft der Hand	1	2	3	4	5	M
5	Schmerzstärke der Hand	1	2	3	4	5	M
6	Sensibilität (das Gefühl) der Hand	1	2	3	4	5	M

6. *Soziale Beeinträchtigungen in der vergangenen Woche*

**Nr**		**Überhaupt nicht**	**Ein bisschen**	**Mäßig**	**Ziemlich**	**Sehr**	Anm
1	Wie schwer haben Beschwerden mit Ihren Händen Ihre sozialen Aktivitäten mit Familie, Freuden oder Vereinen eingeschränkt?	1	2	3	4	5	D
2	Inwieweit waren Sie wegen Beschwerden mit Ihren Händen in der Ausübung Ihrer Berufstätigkeit oder anderer Alltagstätigkeiten beeinträchtigt?	1	2	3	4	5	D
3	Ich empfinde meine Fähigkeiten als eingeschränkt, ich habe weniger Selbstvertrauen oder ich fühle, dass ich mich weniger nützlich machen kann.	1	2	3	4	5	D
4	Ich war zufrieden mit der äußeren Erscheinung (dem Aussehen) meiner Hand.	1	2	3	4	5	M
5	Ich fühlte mich aufgrund des Aussehens meiner Hand in der Öffentlichkeit manchmal unwohl.	1	2	3	4	5	M
6	Das äußere Erscheinungsbild (das Aussehen) meiner betroffenen Hand deprimierte mich.	1	2	3	4	5	M
7	Das äußere Erscheinungsbild (das Aussehen) störte mich in meinen normalen sozialen Aktivitäten.	1	2	3	4	5	M
